# The Role of Multimodality Imaging in Athlete’s Heart Diagnosis: Current Status and Future Directions

**DOI:** 10.3390/jcm10215126

**Published:** 2021-10-31

**Authors:** Antonello D’Andrea, Simona Sperlongano, Vincenzo Russo, Flavio D’Ascenzi, Giovanni Benfari, Francesca Renon, Stefano Palermi, Federica Ilardi, Francesco Giallauria, Giuseppe Limongelli, Eduardo Bossone

**Affiliations:** 1 Division of Cardiology, Department of Traslational Medical Sciences, University of Campania Luigi Vanvitelli, 80131 Naples, Italy; sperlongano.simona@gmail.com (S.S.); v.p.russo@libero.it (V.R.); frances.renon@gmail.com (F.R.); limongelligiuseppe@libero.it (G.L.); 2 Department of Cardiology and Intensive Coronary Care, Umberto I Hospital, 84014 Nocera Inferiore, Italy; stefanopalermi8@gmail.com; 3 Department of Medical Biotechnologies, Division of Cardiology, University of Siena, 53100 Siena, Italy; Flavio.dascenzi@unisi.it; 4 Department of Cardiology, Section of Medicine, University of Verona, 37129 Verona, Italy; giovanni.benfari@univr.it; 5 Department of Advanced Biomedical Sciences, Federico II University Hospital, 80131 Naples, Italy; fedeilardi@gmail.com; 6 Mediterranea Cardiocentro, 80122 Naples, Italy; 7 Department of Translational Medical Sciences, Federico II University of Naples, 80131 Naples, Italy; giallauriafrancesco@gmail.com; 8 Cardiology Division, Department of Internal Medicine, A. Cardarelli Hospital, 80131 Naples, Italy; ebossone@hotmail.com

**Keywords:** athlete’s heart, sudden cardiac death, cardiomyopathies, multimodality imaging, echocardiography, stress echocardiography, strain, cardiac magnetic resonance, cardiac computed tomography

## Abstract

“Athlete’s heart” is a spectrum of morphological and functional changes which occur in the heart of people who practice physical activity. When athlete’s heart occurs with its most marked expression, it may overlap with a differential diagnosis with certain structural cardiac diseases, including cardiomyopathies, valvular diseases, aortopathies, myocarditis, and coronary artery anomalies. Identifying the underlying cardiac is essential to reduce the potential for sudden cardiac death. For this purpose, a spectrum of imaging modalities, including rest and exercise stress echocardiography, speckle tracking echocardiography, cardiac magnetic resonance, computed tomography, and nuclear scintigraphy, can be undertaken. The objective of this review article is to provide to the clinician a practical step-by-step approach, aiming at distinguishing between extreme physiology and structural cardiac disease during the athlete’s cardiovascular evaluation.

## 1. Introduction

The term “athlete’s heart” refers to a spectrum of electrical, morphological, and functional heart adaptations of the heart to athletic training [1,2]. 

When athlete’s heart occurs in its most marked expression, it can overlap with mild phenotypes of cardiac diseases. Therefore, it is important for the clinician to be able to differentiate this extreme form of physiological adaptation from pathology (such as hypertrophic, dilated, arrhythmogenic, and left ventricular (LV) non-compaction cardiomyopathy). For this purpose, different modern imaging modalities, including traditional and novel echocardiographic techniques, cardiac magnetic resonance (CMR), and computed tomography (CT), can be used. Identifying the pathologic cardiovascular condition which may be hidden behind athlete’s heart is crucial to prevent sudden cardiac death [3,4]. 

The objective of this review article is to provide physicians with a practical, diagnostic step-by-step approach aiming at distinguishing between extreme physiology and structural cardiac disease during the cardiovascular evaluation of athletes.

## 2. Clinical and Electrocardiographic Evaluation

Clinical history, physical examination, and the standard 12-lead electrocardiogram (ECG) represent the first step of the athlete’s evaluation. 

Clinical history should be focused on the type and volume of physical activity practiced by the athlete: it is possible to hypothesize an “athlete’s heart” only if training loads are really heavy. Additionally, the use of performance-enhancing drugs should be investigated and taken into account during history collection.

Athlete’s heart is commonly (up to 80%) associated with adaptative ECG changes [5,6]. Sinus bradycardia, first-degree atrioventricular (AV) block, and early repolarization pattern are training-related ECG findings due to increased vagal tone and/or reduced sympathetic activity. Sinus bradycardia <40 beats per minute, Mobitz type 1 second degree AV block, and junctional rhythm are not uncommon and do not warrant further investigation in asymptomatic athletes. Finally, highly trained athletes’ ECGs often exhibit signs of physiologic cardiac chambers’ remodeling, such as pure QRS voltage criteria for LV hypertrophy (e.g., isolated Sokolow–Lyon criterion). Voltage criteria for right ventricular (RV) hypertrophy, right or left axis deviation, right and/or left atrial enlargement require further evaluation just in presence of symptoms or abnormal physical examination. 

Table 1 summarizes all the athletes’ clinical and electrocardiographic features which are not considered training-related or physiologic adaptations to exercise, and therefore require further investigation.

## 3. Echocardiography

According to the current recommendation of the European Association of Preventive Cardiology (EAPC) and the European Association of Cardiovascular Imaging (EACVI), echocardiography is a second-line investigative tool for the differential diagnosis between the athlete’s physiological adaptation to exercise and underlying malignant cardiac conditions [1,7,8]. However, in contrast with contemporary recommendations, standard echocardiography is often used as a first-line screening tool in the pre-participation cardiovascular evaluation of professional and amateur athletes, even in the setting of a normal clinical and electrocardiographic evaluation [7,9]. 

**Figure 1:**
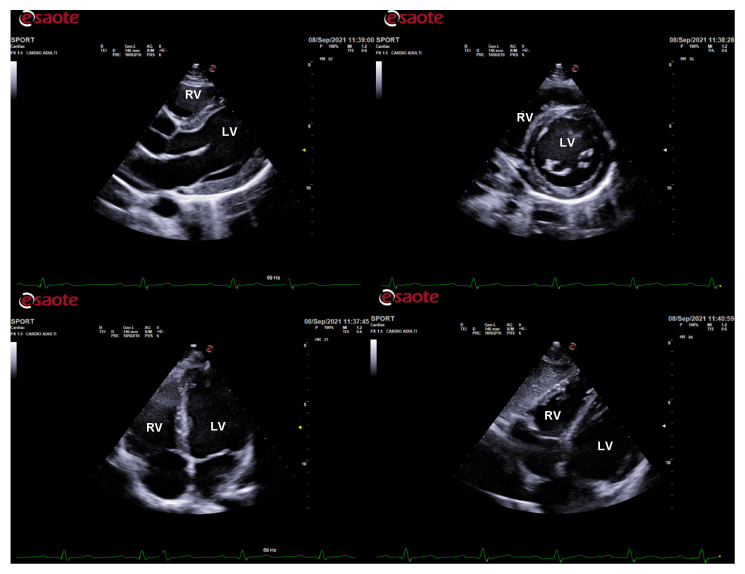
Standard echocardiography in an endurance athlete (cyclist). The physiological hypertrophy of the athlete’s heart is characterized by harmonic and symmetric wall thickening which is homogeneously distributed and involves all the cardiac chambers. In parasternal long-axis (top left), short-axis (top right), apical 4-chamber (bottom left), and subcostal (bottom right) views, both left and right chamber dilatation is evident. LV: left ventricle; RV: right ventricle.

The physiological hypertrophy of athlete’s heart is characterized by a harmonic and symmetric wall thickening that is homogeneously distributed and involves all the cardiac chambers (Figure 1). The exercise-induced chamber thickening is proportional to the type of sport participated (mainly in combined power and endurance disciplines) and it is reversible after temporary (3 months) detraining [10]. A physiological enlargement of both ventricles is usually observed (mostly among endurance athletes), together with a proportional atrial enlargement [11,12,13,14,15,16]. Despite the cardiac chambers’ hypertrophy and enlargement, cardiac systolic function is not altered in athletes, with no significant differences compared to untrained subjects [12,17,18,19]. Likewise, LV diastolic function is normal and an increased contribution of early filling velocity at rest (E/A > 2) can be observed [20,21,22,23,24]. Finally, aortic root diameters are, generally, normal in athletes [25,26,27].

Table 2 reports the echocardiographic features which have to be considered non-physiological in athletes, and thus should raise suspicion of underlying cardiovascular disease.

### 3.1. Exercise Stress Echocardiography 

Exercise stress echocardiography (ESE) is a reliable, safe, non-invasive imaging test, well-tolerated by the athletes, which can be combined with clinical and electrocardiographic information to detect cardiac abnormalities (Table 2).

A typical indication of ESE is the detection of exercise-induced ischemia in athletes with chest pain and/or ECG anomalies, and suspicion of coronary artery disease or congenital coronary artery anomalies [1].

In endurance athletes with LV and/or RV dilatation and mildly reduced ejection fraction at rest, ESE may be used to assess contractile reserve during exercise. A significant improvement in contractility during physical exertion (e.g., ΔLV ejection fraction > 5%) suggests a physiological cardiac remodeling; conversely, an absent or subnormal improvement is in favor of a pathological condition (e.g., dilated cardiomyopathy, LV non-compaction, arrhythmogenic cardiomyopathy) [2,31,32,33]. Similarly, exercise-induced ventricular arrhythmias support the hypothesis of underlying cardiac disease.

An LV outflow tract gradient >50 mmHg during or immediately after exercise in athletes with LV hypertrophy complaining symptoms (syncope, shortness of breath) can be suggestive of hypertrophic cardiomyopathy [1,32].

Finally, ESE may be useful in athletes with valvular heart disease, where it provides information about exercise tolerance, biventricular contractile reserve, changes in hemodynamics (LV filling pressure, pulmonary pressure), and in valvular functional parameters (transvalvular gradients, regurgitation entity) [32].

### 3.2. New Echocardiographic Technologies

Speckle tracking echocardiography (STE) is a relatively recent echocardiographic technique of deformation imaging that has provided new insights into the characterization of the athletes’ myocardial properties [34]. It is able to detect subclinical ventricular systolic function in an early-stage cardiac disease when LVEF is still normal.

LV global longitudinal strain (GLS) obtained by STE is the most used parameter in clinical practice, and it is not significantly different between athletes and healthy controls. Therefore, a reduction in longitudinal strain in athletes has to be considered as a subclinical sign of LV contractile dysfunction and should raise the suspicion of myocardial disease, particularly in the presence of doubtful LV hypertrophy or dilatation [35,36,37,38,39,40,41,42]. A blunted increase in GLS (ΔGLS < 2%) during ESE, meaning a limited contractile reserve, favors the diagnosis of cardiomyopathy rather than athlete’s heart [32].

Data regarding the interpretation of RV, STE-derived parameters in athletes are still controversial [43,44,45,46,47,48,49]. Strenuous and chronic exercise training seems to have a detrimental effect on RV function, with reduction of RV strain immediately after the endurance race, followed by complete recovery [33,50]. Finally, RV strain imaging can be useful to distinguish between physiology and pathology, given its ability in identifying regional wall motion abnormalities in patients with arrhythmogenic cardiomyopathy [51].

During the last decades, STE has also been applied to the evaluation of left and right atrial function [15,46,52,53,54]. In both athlete’s heart and cardiomyopathy, atrial enlargement can be found, but atrial deformation indices are reduced only in the latter. 

**Figure 2:**
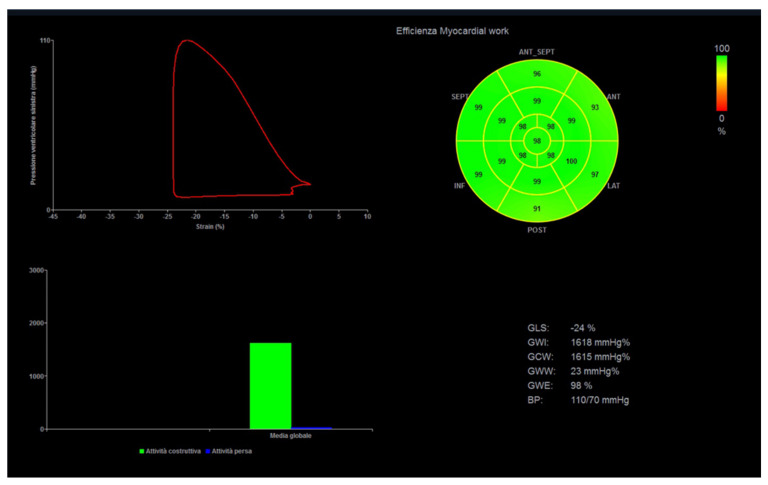
Myocardial work analysis in a power athlete (bodybuilder). On the left, left ventricular pressure–strain loop, showing the relationship between left ventricular systolic pressure and global longitudinal strain. On the right, the 17-segment bull’s-eye representation of myocardial work efficiency, showing homogeneous areas of high efficiency coded in green.

Myocardial work (MW) is a novel, less load-dependent ultrasonographic index of LV contractile function, which corrects STE-derived parameters for afterload, by using systolic blood pressure [55]. In different physiologic and pathologic conditions, an increased afterload may lead to strain impairment, with preserved or increased MW indices. This may be important for athletes with variable blood pressure and loading conditions from exam to exam and in different phases of the training program (Figure 2).

**Figure 3:**
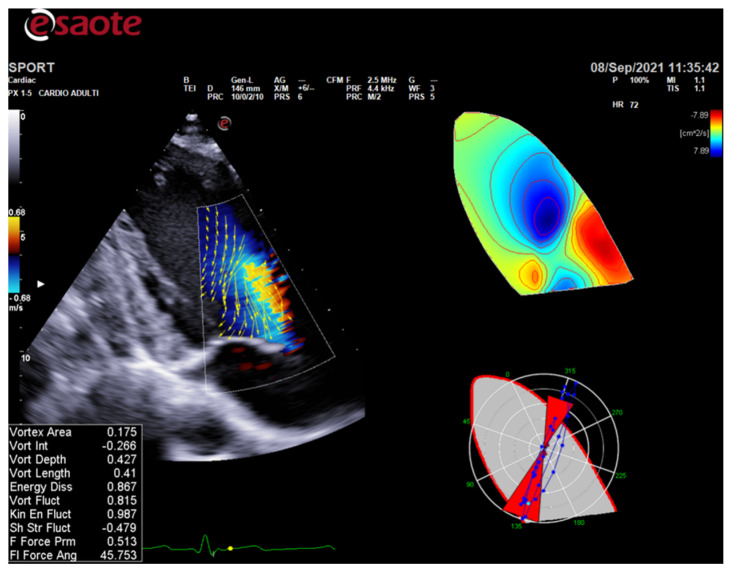
HyperDoppler flow analysis in an endurance athlete. On the left, the flow velocity vector map shows the blood flow that circulates towards the direction of the left ventricular outflow tract. On the right, the circulation parametric map brings to light the formation of a pair of vortices immediately below the aortic valve.

ColorDoppler flow mapping is an advanced echocardiographic tool that evaluates LV function through the analysis of intracardiac flows [56]. LV vortex flow study may provide new insights into the characterization of athlete’s heart properties and its differences with normal subjects and patients with cardiomyopathies (Figure 3).

Finally, three-dimensional (3D) echocardiography improves the diagnostic capability of cardiac ultrasound in evaluating cardiac anatomy, ventricular function, valvular disease, and blood flow velocity. It allows quantifying LV volume and mass, provides data on LV remodeling and function, and can show, in detail, the heart chambers’ morphological features [57].

## 4. Cardiac Magnetic Resonance

Cardiac magnetic resonance (CMR) is the second most valuable imaging method for the differential diagnosis between physiology and pathology in athletes. It can help discriminate health from disease where echocardiography leaves doubts.

**Figure 4:**
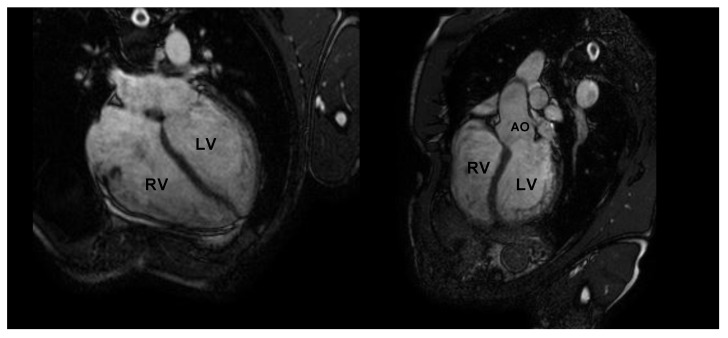
Cardiac magnetic resonance in an endurance athlete (long-distance swimmer). A symmetric dilatation of both right and left ventricular chambers is clearly depicted in 4-chamber (left panel) and 5-chamber (right panel) views. AO: aorta; LV: left ventricle; RV: right ventricle.

**Figure 5:**
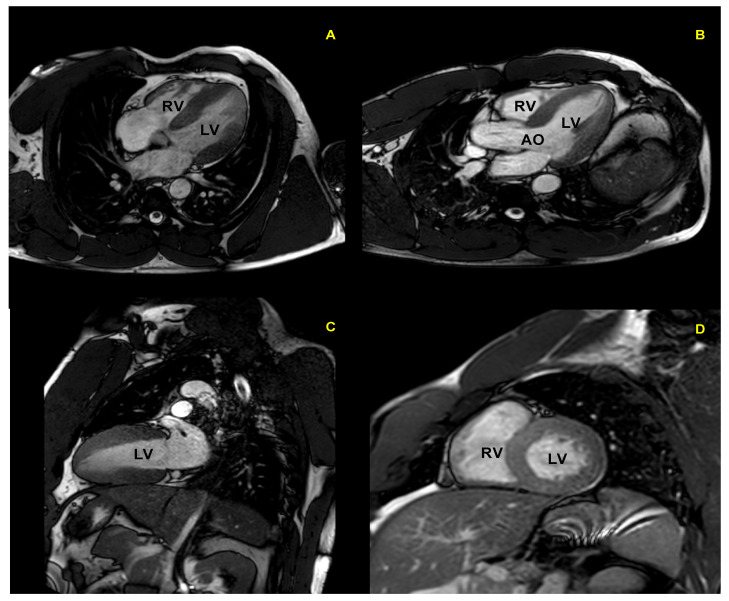
Cardiac magnetic resonance in a top-level power athlete (weightlifter). A significant harmonic and symmetric wall thickening is documented in 4-chamber (**A**), 5-chamber (**B**), 2-chamber (**C**), and short-axis (**D**) views. AO: aorta; LV: left ventricle; RV: right ventricle.

CMR is the gold standard for the definition of myocardial morphology, wall motion assessment, heart chambers size, and tissue characterization. It evaluates, with high accuracy and reproducibility, the heart chambers’ volume and mass, as well as global and regional contractile function [58,59,60]. It is the method of choice for the accurate evaluation of right ventricle morphology and function (Figure 4 and Figure 5).

CMR represents the superior method to identify myocardial fibrosis and its distribution pattern, through the assessment of late gadolinium enhancement (LGE), native T1, and extracellular volume (ECV) mapping [61,62,63,64,65]. Moreover, CMR can identify the presence of edema and fat in myocardial walls. The newer techniques such as LGE, native T1 and ECV mapping (for replacement and diffuse fibrosis), and T2 mapping (for edema) are emerging as valuable tools for the differential diagnosis between athlete’s heart and cardiomyopathies [65,66,67,68]. In particular, the identification of myocardial fibrosis can allow differentiation of athlete’s heart and pathological LV hypertrophy, since fibrosis generally occurs with cardiac remodeling due to pathology [69]. Classically, in hypertrophic cardiomyopathy, mid-wall fibrosis is predominantly found in areas of maximum hypertrophy, although it can be present in non-hypertrophied segments too. 

Myocardial fibrosis has also been observed in athletes with a higher prevalence than in non-athlete healthy populations. In healthy athletes, myocardial fibrosis involves less than 3% of the myocardium. It shows a large variation in quantity, location, and pattern, but it is generally found in the right ventricle (particularly in right ventricle insertion points) or in the interventricular septum. The prevalence of myocardial fibrosis in athlete’s heart appears greater when there is a long-standing history of exercise training, particularly of the endurance type [68]. The prognostic significance of myocardial fibrosis in athlete’s heart is unknown. As for diffuse interstitial fibrosis, studies comparing ECV in athletes and controls have reported similar or lower values of ECV in athletes [70,71].

Heart valve morphology and function, and great vessel structure can be well evaluated by CMR. 

Coronary magnetic resonance angiography shows high accuracy for the non-invasive detection and definition of anomalous coronary arteries, thanks to its ability in identifying the proximal coronary course and generating tomographic images in any orientation [72]. 

Stress CMR, usually with exercise, can be used to identify a reduced functional reserve and early-stage cardiomyopathy when resting functional assessment is mildly abnormal [73]. However, further studies are necessary to evaluate the cost-effectiveness of stress CMR imaging in this setting.

Finally, the possible role of CMR-based deformation imaging in distinguishing athlete’s heart from pathological phenotypes is being studied in different disease settings [66,74,75].

Table 3 summarizes the CMR features of the athletes which orient towards underlying cardiovascular disease.

Limitations of CMR include high cost, less availability than echocardiography, incompatibility with some metallic devices (though this is rarely an issue in athlete populations), the need for evaluation of renal function prior to administration of gadolinium, and claustrophobia.

## 5. Cardiac Computed Tomography and Other Imaging Modalities

**Figure 6:**
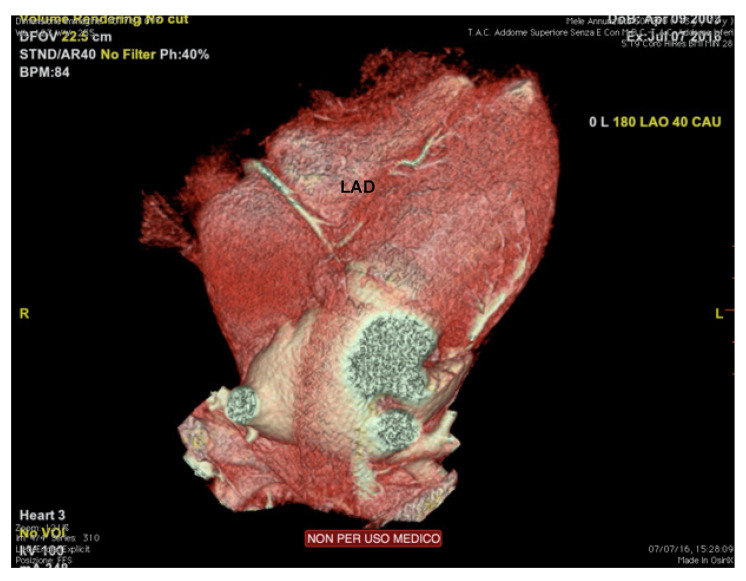
Cardiac computed tomography of an endurance athlete (runner) symptomatic for syncope. The figure shows an intramural course of the left anterior descending coronary artery. LAD: left anterior descending.

Cardiac computed tomography (CCT) shows high accuracy in the evaluation of coronary arteries’ origin, course (including intramural), and termination [1] (Figure 6). Therefore, the main indication to perform a coronary CT angiography (CTA) is the suspicion of coronary artery anomalies (generally under 35 years of age) or atherosclerotic coronary artery disease (over 35), raising from clinical markers (exertional syncope, angina, or arrhythmias), or abnormal exercise test [76].

All athletes with indeterminate, suspected, or confirmed anomalous coronary artery anatomy following echocardiography should undergo CTA or CMR, according to institutional preferences and expertise.

In the case of suspected coronary atherosclerotic disease, CTA is a valuable tool for coronary artery calcium scoring and non-invasive coronary angiography.

If dilatation of aortic root or ascending aorta is suspected or confirmed, at least one comprehensive aortic tomographic assessment (with CTA or CMR according to preference and local expertise) is indicated [76].

Recent technological advances have extended the role of CCT beyond coronary and great vessels evaluation. Morphological and functional assessment of the left ventricle is performed using a retrospective ECG-gated scanning protocol. The evaluation of LV volumes, stroke volume, ejection fraction, and mass have shown excellent correlation with CMR assessment [77].

LV functional evaluation by CCT is particularly useful in claustrophobic patients, unable to undergo CMR, or if contraindications to CMR exist (albeit rare among athletes). Otherwise, CCT cannot be recommended as a first-line imaging technique for functional evaluation of the LV in athletes, given the higher radiation exposure required [73,77].

The use of iodine contrast medium allows the evaluation of ECV and myocardial fibrosis (with the analysis of late iodine enhancement, LIE) by CCT, even if not routinely used in clinical practice, with good agreement with the same evaluations performed by contrast-enhanced CMR [73].

Nuclear scintigraphy can be used for the research of exercise-induced ischemia—as an alternative or integration to ESE—when coronary disease is suspected, with the same indications as in the non-athlete population. 

## 6. Conclusions

A broad spectrum of imaging modalities is available for pre-participation cardiovascular evaluation of the athletes. The clinician should adopt a step-by-step approach, with the aim to distinguish between the physiologic, exercise-induced remodeling and structural cardiac disease. This practical approach is based on the periodic use of first-line screening tools (clinical history, physical examination, and ECG), possibly integrated by standard echocardiography, which is safe and not very time-consuming. Third-line imaging modalities (e.g., stress echocardiography, CMR, cardiac CT, etc.) should be selected when the previous evaluation is still doubtful. Speckle tracking echocardiography provides new insights into the characterization of the athlete’s myocardial properties. However, further accumulation of evidence is needed to refine STE diagnostic performance and make it part of the standard diagnostic workup.

## Figures and Tables

**Table 1:** Athlete’s clinical and electrocardiographic features requiring further investigation.

**Clinical History**
Family history of sudden cardiac death/cardiomyopathies
Palpitations
Chest pain
Syncope
**Physical Examination**
Cardiac murmurs/abnormal cardiac sounds
Marfanoid habitus
**12-Lead ECG**
T-wave inversion *
ST-segment depression
Pathologic Q-waves
Complete LBBB Profound (QRS ≥140 ms) non-specific intraventricular conduction delay
RBBB + left anterior hemiblock ** Epsilon wave Ventricular pre-excitation
Long QTc (≥470 ms in men, ≥480 ms in women)
Type 1 Brugada pattern *** Sinus bradycardia <30 beats/min Sinus pauses ≥3 s First degree AV block ≥400 ms Mobitz type 2 second degree AV block Third-degree AV block Atrial tachyarrhythmias Ventricular arrhythmias PVCs ≥ 2 per 10 s tracing

AV: atrioventricular; ECG: electrocardiogram; LBBB: left bundle branch block; PVC: premature ventricular contraction; RBBB: right bundle branch block; * The juvenile ECG pattern (T-wave inversion in leads V1–V3) can be considered normal up to 16 years of age; after that it warrants further assessment in Caucasian athletes; ** Isolated RBBB does not require further investigation in asymptomatic athletes, with QRS duration <140 ms, and without significant repolarization abnormalities; *** Type 2 Brugada ECG pattern does not require further evaluation unless there is history of syncope or relevant family history.

**Table 2:** Athlete’s echocardiographic findings suggestive of cardiovascular disease.

Differential Diagnosis	Echocardiographic Features
HCM	− LV wall thickness >16 mm *
	− Segmental LV hypertrophy/bizarre LV hypertrophy pattern
	− LV hypertrophy with normal or reduced cavity size (<54 mm)/concentric pattern of hypertrophy
	− Unchanged LV wall thickness with detraining
	− LV systolic dysfunction (reduced strain values)
	− LV diastolic dysfunction (E/A < 1, reduction of e’ velocity on TDI)
	− Abnormal LA enlargement (disproportionate to LV remodeling)
	− Mitral valve anomalies (elongation of mitral chordae, SAM)
	− LV outflow tract obstruction at rest or during exercise
DCM	− LV end-diastolic diameter >60 mm **
	− LV systolic dysfunction (LVEF < 50%)
	− Absent or subnormal LV systolic function improvement during exercise
Myocarditis	− LV dilatation with reduced wall thickness − Increased myocardial wall thickness (myocardial edema) − Global or regional LV systolic dysfunction − Pericardial effusion
LVNC	− LV hypertrabeculation/deep inter-trabecular recesses
	− NC/C layer ratio > 2 in systole/reduced thickness of compact layer (<8 mm in systole)
	− LV systolic dysfunction (LVEF < 50%)
	− Absent or subnormal LV systolic function improvement during exercise
	− LV diastolic dysfunction (mean e’ < 9 cm/s)
AC	− RV dilatation (asymmetric, mainly involving RV outflow tract) ***
	− RV regional wall motion abnormalities (akinesia, dyskinesia, aneurysm)
	− RV systolic dysfunction (FAC ≤ 33%, reduced S’ wave)
	− Absent or subnormal RV systolic function improvement during exercise
	− Unchanged RV size with detraining
BAV	− Presence of two leaflets, with or without raphe, leaflets’ systolic doming − Eccentric diastolic closure of the valve − Aortic root enlargement (>40 mm in M, >34 mm in F) − Larger dimensions of ascending aorta and aortic arch − Aortic dissection − Aortic valve dysfunction (stenosis and/or regurgitation) − LV enlargement − Coartation of the aorta
MVP	− Systolic displacement of one or both MV leaflets, with or without leaflets’ thickening − Mitral regurgitation − LA/LV enlargement
Coronary artery anomalies	− Anomalous origin/proximal course of left main or right coronary artery − LV systolic dysfunction − Regional LV wall motion abnormalities − Exercise-induced LV wall motion abnormalities

AC: arrhythmogenic cardiomyopathy; BAV: bicuspid aortic valve; DCM: dilated cardiomyopathy; F: females; FAC: fractional area change; GLS: global longitudinal strain; HCM: hypertrophic cardiomyopathy; LA: left atrial; LV: left ventricular; LVEF: left ventricular ejection fraction; LVNC: left ventricular non-compaction; M: males; MV: mitral valve; MVP: mitral valve prolapse; NC/C: non-compacted to compacted; RV: right ventricular; SAM: systolic anterior motion; TDI: tissue Doppler imaging; * LV wall thickness ranging from 13 mm and 16 mm is considered a gray zone of LV hypertrophy which overlaps mild HCM [11,28]; ** LV end-diastolic diameter >60 mm can be normal, above all among endurance athletes, in the presence of preserved ejection fraction and normal stroke volume; nevertheless, some endurance athletes can still have lower ejection fraction values (45–50%); *** RV dilatation is common in athletes, particularly endurance disciplines. Therefore, it may be considered suggestive of AC in athletes only when RV size exceeds the major 2010 Task Force Criteria for AC [29,30].

**Table 3:** Athlete’s CMR findings suggestive of cardiovascular disease.

Differential Diagnosis	CMR Features
HCM	− Asymmetric distribution of myocardial hypertrophy, relative apical hypertrophy − RV involvement − Patchy mid-wall pattern of LGE in areas of hypertrophy and at the anterior and posterior RV insertion points − High native myocardial T1 − High ECV
DCM	− Unbalanced LV dilatation − Reduced LV systolic function, low SV − Regional wall motion abnormalities − Positive LGE in dilated left ventricle − High native myocardial T1 − High ECV
Myocarditis	− Myocardial edema − Hyperemia and capillary leak − Positive LGE with mid-wall or subepicardial distribution − Regional or global LV wall motion abnormalities − Pericardial effusion
LVNC	− Unbalanced LV dilatation − NC/C layer ratio > 2.3 in diastole − Reduced LV systolic function − Positive LGE
AC	− RV dilatation *
	− RV/LV RWMA (akinesia, dyskinesia, aneurysm)
	− RV systolic dysfunction (RVEF < 45%) − Presence of LGE with subepicardial and mid-wall distribution also or exclusively in the left ventricle − RV/LV fat
BAV	− Aortic root, ascending aorta, and aortic arch dilatation − Coartation of the aorta

AC: arrhythmogenic cardiomyopathy; BAV: bicuspid aortic valve; CMR: cardiac magnetic resonance; ECV: extracellular volume fraction; HCM: hypertrophic cardiomyopathy; LGE: late gadolinium enhancement; LVNC: left ventricular non-compaction; NC/C: non-compacted to compacted; RV: right ventricular; RVEF: right ventricular ejection fraction; SV: stroke volume; * RV dilatation is common in athletes, particularly endurance disciplines. Therefore, it may be considered suggestive of AC in athletes only when RV size exceeds the major 2010 Task Force Criteria for AC [29,30]. In male athlete’s heart, reference values for RV volume by D’Ascenzi et al. [58] may be used in the differential diagnosis of physiologic vs. pathologic RV dilatation.
